# The way forward to achieve high COVID-19 vaccination and revaccination coverage in a city amid a period of tranquility

**DOI:** 10.3389/fpubh.2022.935243

**Published:** 2022-09-14

**Authors:** Kin On Kwok, Kin Kit Li, Cyrus Lap Kwan Leung, Arthur Tang, Emily Ying Yang Chan, Margaret Ting Fong Tsoi, Wan In Wei, Edward B. McNeil, Samuel Yeung Shan Wong

**Affiliations:** ^1^JC School of Public Health and Primary Care, The Chinese University of Hong Kong, Hong Kong Special Administrative Region, Hong Kong, China; ^2^Stanley Ho Centre for Emerging Infectious Diseases, The Chinese University of Hong Kong, Hong Kong Special Administrative Region, Hong Kong, China; ^3^Hong Kong Institute of Asia-Pacific Studies, The Chinese University of Hong Kong, Hong Kong Special Administrative Region, Hong Kong, China; ^4^Shenzhen Research Institute of the Chinese University of Hong Kong, Shenzhen, China; ^5^Department of Social and Behavioural Sciences, City University of Hong Kong, Hong Kong, China; ^6^College of Computing and Informatics, Sungkyunkwan University, Seoul, South Korea; ^7^Collaborating Centre for Oxford University and CUHK for Disaster and Medical Humanitarian Response, Hong Kong, China; ^8^Nuffield Department of Medicine, University of Oxford, Oxford, United Kingdom; ^9^GX Foundation, Hong Kong, China; ^10^Accident & Emergency Medicine Academic Unit, The Chinese University of Hong Kong, Prince of Wales Hospital, Hong Kong, China

**Keywords:** boosting uptake coverage, COVID-19, Hong Kong, latent profile analysis, vaccine hesitancy

## Abstract

**Background:**

Amid the current COVID-19 pandemic, there is an urgent need for both vaccination and revaccination (“boosting”). This study aims to identify factors associated with the intention to receive a booster dose of the coronavirus (COVID-19) vaccine among individuals vaccinated with two doses and characterize their profiles in Hong Kong, a city with a low COVID-19 incidence in the initial epidemic waves. Among the unvaccinated, vaccination intention is also explored and their profiles are investigated.

**Methods:**

From December 2021 - January 2022, an online survey was employed to recruit 856 Hong Kong residents aged 18 years or over from an established population-based cohort. Latent class analysis and multivariate logistic regression modeling approaches were used to characterize boosting intentions.

**Results:**

Of 638 (74.5%) vaccinated among 856 eligible subjects, 42.2% intended to receive the booster dose. Four distinct profiles emerged with believers having the highest intention, followed by apathetics, fence-sitters and skeptics. Believers were older and more likely to have been vaccinated against influenza. Older age, smoking, experiencing no adverse effects from a previous COVID-19 vaccination, greater confidence in vaccines and collective responsibility, and fewer barriers in accessing vaccination services were associated with higher intentions to receive the booster dose. Of 218 unvaccinated, most were fence-sitters followed by apathetics, skeptics, and believers.

**Conclusion:**

This study foretells the booster intended uptake lagging initial vaccination across different age groups and can help refine the current or future booster vaccination campaign. Given the fourth COVID-19 vaccine dose may be offered to all adults, strategies for improving boosting uptake include policies targeting young adults, individuals who experienced adverse effects from previous doses, fence-sitters, apathetics, and the general public with low trust in the health authorities.

## Introduction

Following the first detection of the SARS-CoV-2 (B.1.1.529) variant in Gauteng Province, South Africa, Omicron has replaced Delta to become the most dominant COVID-19 variant in many populations globally. Given its high transmissibility and vaccine breakthrough infection nature (1), the substantial infection posed a threat to the unvaccinated, a particularly vulnerable group. Vaccination is still considered to effectively protect vulnerable groups against severe illness, hospitalization and mortality associated with infection triggered by Omicron (2). A recent study revealed that 20% of those receiving two doses of BNT162b2 had detectable neutralizing antibodies against Omicron at 56 days after the first dose (3). Receiving a three-dose short course of BNT162b2 vaccine can strengthen the vaccine effectiveness (VE) against symptomatic infection and severe outcomes (hospitalization or death) due to both the Omicron and Delta variants (4). This supports the urgent need for both vaccination and revaccination (“boosting”).

Hong Kong, a densely populated city with frequent daily social contacts and an important travel hub for foreign multinationals, has experienced 3 major epidemic waves with a low cumulative number of confirmed cases of under 12,000 from January 2020 to November 2021 (5). A territory-wide vaccination campaign began in February 2021 in which two choices of vaccine, namely BNT162b2 (BioNTech hereafter) and CoronaVac (Sinovac hereafter), were provided (6). In terms of speed, it took 9 months to get 70% of the eligible population vaccinated with the first dose (7), which was longer than the United Kingdom and Israel (7) while mainland China fully vaccinated its entire population within 3 months (8). The persistence of vaccine hesitancy, a phenomenon whereby people are reluctant to receive the vaccine or delay their decision to accept it despite the availability of vaccine services (9), among the unvaccinated, and receipt of a booster dose lagging the initial vaccination (10), increased the difficulties in administering the next phase of the vaccination campaign. Given that more than 6 months have passed since the majority of Hong Kong citizens had received their first vaccine dose (6), an unprecedented revaccination campaign in both speed and scale should be implemented as soon as possible.

Different types of vaccine hesitancy may be sensitive to different promotional strategies. To enhance the community protection against infection and severe outcomes with the Omicron variant and a potential transition period before the establishment of regular infection seasonality, unraveling the vaccine hesitancy profiles of the general population and identifying determinants of intention to be revaccinated is essential for refining the overall strategy of current and future booster vaccination campaigns. In this study, we report a cross-sectional analysis of a representative population survey of adults residing in Hong Kong to first determine, among those who have received or will receive their second COVID-19 vaccine dose, the proportion who intend to receive a third (booster) dose. Second, we determine factors associated with the intention to receive a booster dose and present the profiles among them. Third, we explore the proportion of unvaccinated and identify their profiles.

## Methods

### Subject recruitment

A community cohort was set up within 36 h after the first reported COVID-19 case in Hong Kong in January 2020 to longitudinally assess the risk perception toward COVID-19 among the general population (11). District councilors distributed an online survey link *via* the routes in which they normally disseminate information to their target residents. Our cohort comprised citizens from all administrative districts in Hong Kong, and thus was representative of the Hong Kong population. The cohort was maintained with 9 follow-up rounds and all subjects in the existing cohort were invited to join the study. Additional subjects were recruited with the same sampling methodology from December 2021 - January 2022.

### Eligibility criteria

Eligibility criteria included being aged 18 years or more and a resident of Hong Kong, defined as living in Hong Kong for at least 5 days per week on average during the past month. We included two groups of people: those who were unvaccinated (hereafter unvaccinated) and those who had received or intended to receive two doses (hereafter vaccinated). Thus, we excluded those who received only one dose and had no intention to receive further doses.

### Outcome

The primary outcome was the intention to receive a third (booster) dose among the vaccinated, which was assessed with the question: “How likely will you receive the third dose of the COVID-19 vaccine?” Responses were rated on an 11-point Likert scale ranging from 0 (absolutely unlikely) to 10 (absolutely likely). Those who had already received their third dose were given a score of 10. The secondary outcome, intention to receive the first vaccine dose among the unvaccinated, was evaluated by rating: “How likely will you receive the COVID-19 vaccine?” and rated on the same 11-point Likert scale described above.

### Covariates

Independent variables included socio-demographic characteristics such as age, sex, and employment status; health-related information such as smoking status, long-term illnesses, influenza vaccination history, history of organ transplant, and treatment for immunosuppression; and adverse effects experienced from any of their previous COVID-19 vaccine doses. The 15-item scale of 5C psychological antecedents to vaccination (12) was used as a measurement of the indicators in the latent profile analysis., which covered five different domains of psychological factors determining vaccine hesitancy, namely confidence (trust in vaccine effectiveness, safety, and necessity and the system that delivers it), complacency (perceived the disease as low risk), constraints (perceived low vaccine availability, affordability, and accessibility), calculation (engagement in information searching), and collective responsibility (willingness to protect others via herd immunity). Responses to the items ranged from 1 (strongly disagree) to 7 (strongly agree) with 4 representing a neutral viewpoint.

### Statistical analysis

To examine profiles and factors associated with the intention to receive the COVID-19 vaccine, two frameworks were adopted. We first analyzed vaccination intention as a continuous variable in a latent profile analysis (LPA) framework. Second, we defined those expressing a level of intention >6 as having an intention to receive the third vaccine dose in a multivariate logistic regression framework.

(i) Person-centered approach

LPA was employed to identify latent subgroups within the full sample based on the 5C psychological antecedents of vaccine hesitancy (13). The number of subgroups was determined based on prior findings (14) and several statistics for model comparison and interpretability (15). We modeled the predictors and vaccination intentions for two subgroups - one each for the vaccinated and unvaccinated subgroups. The profile structure of the full sample was adopted to each subgroup by fixing the profile means for each of the 5C indicators.

(ii) Variable-centered approach

To determine factors independently associated with the intention to receive the third COVID-19 vaccine dose among the vaccinated subgroup, we fitted an initial multivariate logistic regression model to the data in which mildly significant (*p*-value < 0.1) variables from the univariate analysis were included. A stepwise backward elimination technique was then used to reduce the complexity of the model based on the AIC value. Variables with a *p*-value < 0.05 were included in the final multivariate model. The strength of association for each risk factor in the final model was presented as an adjusted odds ratio (OR) with 95% confidence interval (CI). We also performed a sensitivity analysis to determine whether including participants who gave invalid utility scores changed the results.

All analyses were conducted in R v4.1.0 (16) and Mplus v7.4 (17).

### Ethics

The study was approved by the Survey Behavioral Research Ethics Committee of The Chinese University of Hong Kong (reference number: SBRE-20-037).

## Results

### Respondent characteristics

Of the 862 eligible respondents, 644 (74.7%) had received at least one COVID-19 vaccine dose (611 (70.9%) received two doses) and the remaining 218 (25.3%) had not received any. Of the 33 who had received only one dose, six were excluded from the analysis because they indicated they would not receive a second dose (two gave multiple reasons; two stated their doctors did not recommend it; one stated they had already been infected with COVID-19; one thought a single dose was sufficient). Therefore, 856 eligible responses were analyzed. The median (IQR) survey completion time among all eligible participants was 43 (interquartile range: 30–65) min. A summary of the variables and comparison of vaccination intention within the vaccinated and unvaccinated subgroups are presented in Table 1. Descriptive results are reported in the Appendix.

**Table 1:** Characteristics of 856 respondents by vaccinated (n = 638) and unvaccinated groups (n = 218).

	**Total** ** (*N* = 856)**	**Vaccinated (*****n*** = **638)**			**Unvaccinated (n** = **218)**		
**Factors**		**No intention ** ** (*n* = 369)**	**Intention (*n* = 269)**	***P*-value^2^**	**No intention (*n* = 199)**	**Intention** **(*n* = 19)**	***P*-value^2^**
**Age group**				**<** **0.001**			0.448
18–24	61 (9.6)	44 (72.1)	17 (27.9)		20 (90.9)	2 (9.1)	
25–34	199 (31.2)	136 (68.3)	63 (31.7)		68 (89.5)	8 (10.5)	
35–44	194 (30.4)	108 (55.7)	86 (44.3)		57 (91.9)	5 (8.1)	
45–54	105 (16.5)	49 (46.7)	56 (53.3)		27 (100.0)	0 (0.0)	
55–64	58 (9.1)	27 (46.6)	31 (53.4)		19 (86.4)	3 (13.6)	
65+	21 (3.3)	5 (23.8)	16 (76.2)		8 (88.9)	1 (11.1)	
**Sex**				0.393			0.793
Female	238 (37.3)	132 (55.5)	106 (44.5)		72 (90.0)	8 (10.0)	
Male	400 (62.7)	237 (59.2)	163 (40.8)		127 (92.0)	11 (8.0)	
**Cohort**				**<** **0.001**			0.6
First (original)	199 (31.2)	136 (68.3)	63 (31.7)		70 (93.3)	5 (6.7)	
Second (top-up)	439 (68.8)	233 (53.1)	206 (46.9)		129 (90.2)	14 (9.8)	
**Employed full-time**				**0.045**			1
Yes	259 (40.6)	137 (52.9)	122 (47.1)		85 (91.4)	8 (8.6)	
No	379 (59.4)	232 (61.2)	147 (38.8)		114 (91.2)	11 (8.8)	
**Smoking status**				0.292			0.129
Regular	30 (4.7)	13 (43.3)	17 (56.7)		11 (100.0)	0 (0.0)	
Occasional	18 (2.8)	9 (50.0)	9 (50.0)		4 (100.0)	0 (0.0)	
Former	14 (2.2)	7 (50.0)	7 (50.0)		4 (66.7)	2 (33.3)	
Non-smoker	576 (90.3)	340 (59.0)	236 (41.0)		180 (91.4)	17 (8.6)	
**Smoking status**				0.085			1
Smoker (incl. former)	62 (9.7)	29 (46.8)	33 (53.2)		19 (90.5)	2 (9.5)	
Non-smoker	576 (90.3)	340 (59.0)	236 (41.0)		180 (91.4)	17 (8.6)	
**Perceived health**							1
Very bad	7 (1.1)	7 (100.0)	0 (0.0)		5 (100.0)	0 (0.0)	
Bad	18 (2.8)	10 (55.6)	8 (44.4)		6 (100.0)	0 (0.0)	
Average	154 (24.1)	95 (61.7)	59 (38.3)		59 (92.2)	5 (7.8)	
Good	367 (57.5)	209 (56.9)	158 (43.1)		100 (90.1)	11 (9.9)	
Very good	92 (14.4)	48 (52.2)	44 (47.8)		29 (90.6)	3 (9.4)	
**Food/drug allergies**							0.575
Yes	94 (14.7)	54 (57.4)	40 (42.6)		43 (89.6)	5 (10.4)	
No	544 (85.3)	315 (57.9)	229 (42.1)		156 (91.8)	14 (8.2)	
**Long-term illnesses** ^**1**^				0.166			0.867
Yes	275 (43.1)	150 (54.5)	125 (45.5)		95 (90.5)	10 (9.5)	
No	363 (56.9)	219 (60.3)	144 (39.7)		104 (92.0)	9 (8.0)	
**Received immunosuppressant treatment**				0.75			1
Yes	10 (1.6)	5 (50.0)	5 (50.0)		7 (100.0)	0 (0.0)	
No	628 (98.4)	364 (58.0)	264 (42.0)		192 (91.0)	19 (9.0)	
**Respiratory symptoms (past 2 weeks)**				0.703			1
Yes	59 (9.2)	36 (61.0)	23 (39.0)		20 (90.9)	2 (9.1)	
No	579 (90.8)	333 (57.5)	246 (42.5)		179 (91.3)	17 (8.7)	
**Influenza vaccination (previous season)**				**<** **0.001**			**0.003**
Yes	198 (31.0)	93 (47.0)	105 (53.0)		26 (76.5)	8 (23.5)	
No	440 (69.0)	276 (62.7)	164 (37.3)		173 (94.0)	11 (6.0)	
**Influenza vaccination (current season)**				**<** **0.001**			**<0.001**
Yes	115 (18.0)	44 (38.3)	71 (61.7)		14 (66.7)	7 (33.3)	
No	523 (82.0)	325 (62.1)	198 (37.9)		185 (93.9)	12 (6.1)	
**Experienced adverse effects from the 1**^**st**^ **COVID dose**				**<** **0.001**	
Yes	354 (55.5)	235 (66.4)	119 (33.6)		
No	284 (44.5)	134 (47.2)	150 (52.8)		
**Experienced adverse effects from the 2**^**nd**^ **COVID dose**				**<** **0.001**	
Yes	379 (62.0)	242 (63.9)	137 (36.1)		
No/not applicable	232 (38.0)	104 (44.8)	128 (55.2)		
**Vaccine hesitancy constructs** ^3^							
Confidence	4.3 (3.3–5.3)	4.0 (3.3–5.0)	5.3 (4.0–6.0)	**<** **0.001**	3.7 (2.0–4.7)	4.3 (3.0–5.2)	**0.027**
Complacency	4.0 (3.0–4.3)	4.0 (3.0–4.3)	3.3 (2.3–4.0)	**<** **0.001**	4.0 (3.3–4.7)	3.7 (2.2–4.0)	**0.002**
Constraint	3.3 (2.0–4.0)	3.7 (2.7–4.0)	2.7 (2.0–4.0)	**<** **0.001**	3.7 (2.7–4.0)	3.3 (2.0–4.2)	0.35
Calculation	5.7 (4.7–6.0)	5.3 (4.3–6.0)	5.7 (4.7–6.0)	0.173	6.0 (5.0–6.7)	6.0 (5.0–6.3)	0.981
Collective	4.7 (4.0–6.0)	4.7 (4.0–5.7)	5.7 (4.7–6.3)	**<** **0.001**	4.0 (3.3–4.7)	4.7 (3.7–5.3)	0.215

^1^Includes ear/nose/throat conditions such as rhinitis, hay fever, and tinnitus; hypertension; kidney disease; skin conditions such as eczema and psoriasis; depression; tuberculosis; inherited blood disorders (e.g., thalassemia, hemophilia); eye diseases (e.g., glaucoma, cataract, vision loss/blindness); hypercholesterolemia; diabetes mellitus; and other miscellaneous illnesses.

^2^The chi-squared or Fisher's exact test was used to assess the crude association between categorical covariates and the outcomes as appropriate while the Wilcoxon rank-sum test was used for continuous variables.

^3^The level of agreement to each statement of the 5C vaccine hesitancy scale ranged from 1 to 7 (Figure 2).

Bold figures indicate *p*-values smaller than 0.05.

(i) Full sample

**Figure 1:**
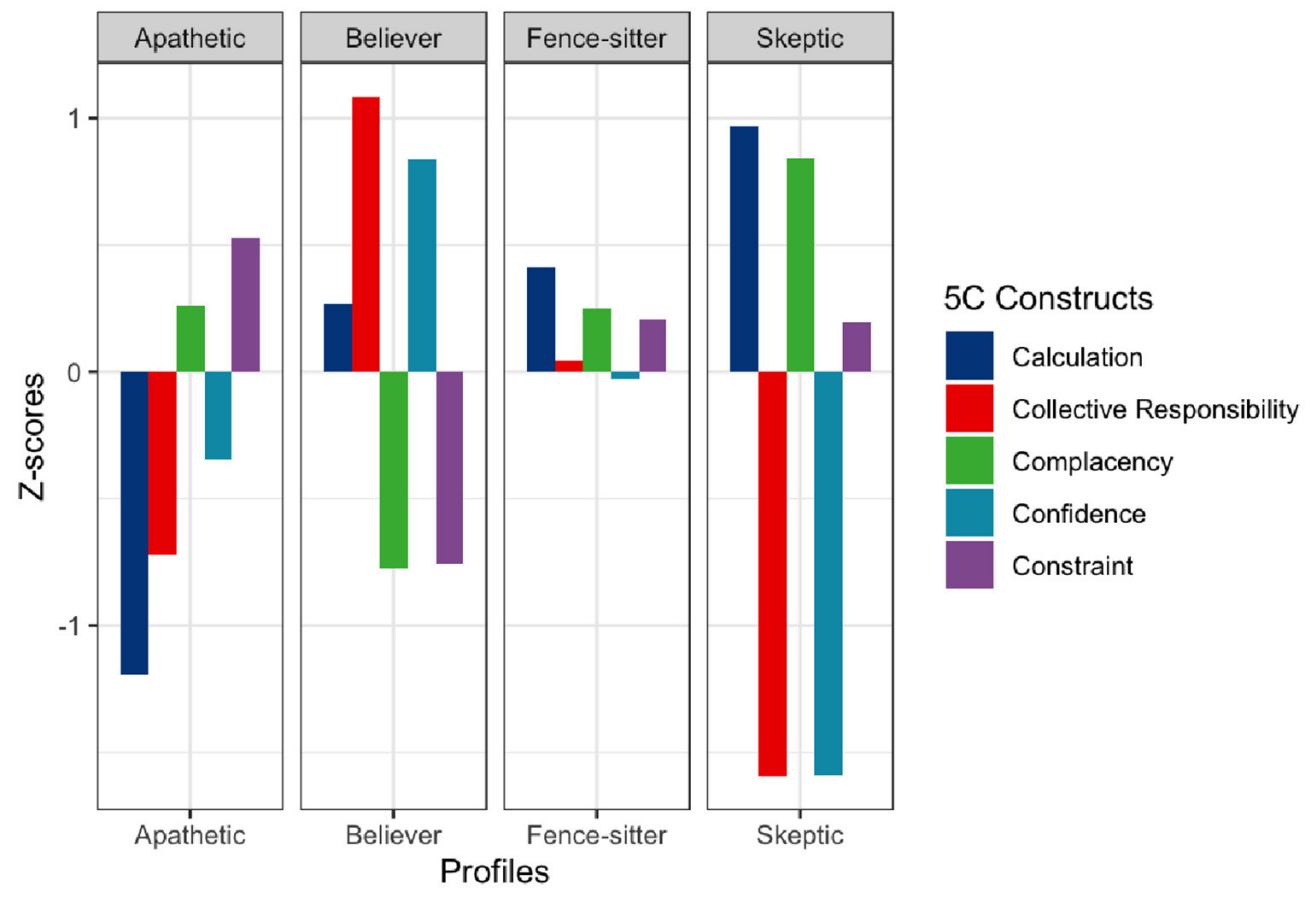
5C characteristics of the 4-profile solution (*N* = 856).

In the LPA on 856 eligible participants, we arrived at a 4-profile solution (Table 2, Figure 1, and Supplementary Table 1). These profiles were labeled “believers” (30.1%), “fence-sitters” (34.0%), “apathetics” (26.3%), and “skeptics” (9.6%). Based on mean standardized (z) scores, believers exhibited high confidence (z = 0.84), high collective responsibility (z = 1.08), low complacency (z = −0.78), and low constraint (z = −0.76). Fence-sitters had average scores in all constructs while apathetics scored low in calculation (z = −1.19) but high in complacency (z = 0.25) and constraint (z = 0.52). In contrast, skeptics were characterized by having low confidence (z = −1.58), low collective responsibility (z = −1.57), high complacency (z = 0.84), and high constraint (z = 0.19) (Supplementary Table 2).

**Table 2:** Descriptive information of the four latent profiles (N = 856).

		**5C Constructs**										**Intention to receive the COVID-19 vaccine**							
		**Confidence**		**Complacency**		**Constraint**		**Calculation**		**Collective responsibility**		**First dose** **(*****n*** = **218)**				**Third dose** **(*****n*** = **638)**			
**Profile**	***n* (%)**	**Mean**	**95% CI**	**Mean**	**95% CI**	**Mean**	**95% CI**	**Mean**	**95% CI**	**Mean**	**95% CI**	**No** ***n*** **(%)**		**Yes** ***n*** **(%)**		**No** ***n*** **(%)**		**Yes** ***n*** **(%)**	
Skeptic	82 (9.6)	1.94	1.65, 2.23	4.59	4.17, 5.01	3.52	3.11, 3.92	6.42	6.26, 6.57	2.97	2.65, 3.29	56	(28.1%)	2	(10.5%)	22	(6.0%)	2	(0.7%)
			(−1.58)		(0.84)		(0.19)		(0.97)		(−1.57)	
Believer	258 (30.1)	5.47	5.18, 5.77	2.68	2.25, 3.11	2.30	2.03, 2.57	5.76	5.61, 5.90	6.04	5.88, 6.21	18	(9.0%)	5	(26.3%)	86	(23.3%)	149	(55.4%)
			(0.84)		(−0.78)		(−0.76)		(0.27)		(1.08)	
Fence-sitter	291 (34.0)	4.27	3.91, 4.64	3.89	3.68, 4.10	3.47	3.13, 3.80	5.84	5.72, 5.96	4.91	4.52, 5.30	72	(36.2%)	5	(26.3%)	145	(39.3%)	69	(25.7%)
			(−0.02)		(0.24)		(0.20)		(0.42)		(0.05)	
Apathetic	225 (26.3)	3.81	3.64, 3.97	3.91	3.80, 4.02	3.87	3.73, 4.00	4.26	4.06, 4.46	4.04	3.94, 4.13	53	(26.6%)	7	(36.8%)	116	(31.4%)	49	(18.2%)
			(−0.34)		(0.25)		(0.52)		(−1.19)		(−0.71)	

Numbers in the brackets represent the Z-scores.

**Figure 2:**
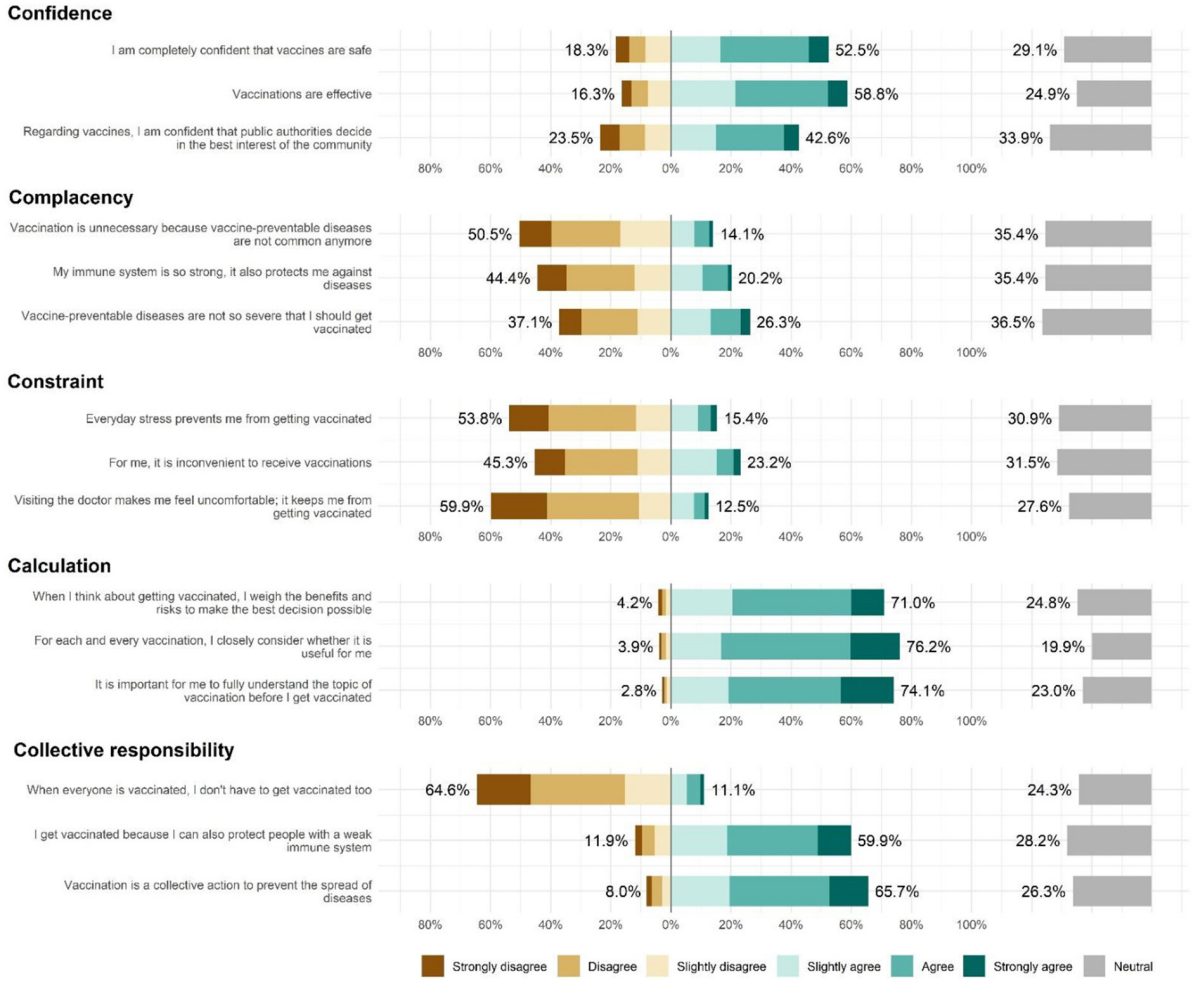
Distributions in the level of agreement to each statement of the 5C vaccine hesitancy scale.

(ii) Vaccinated subgroup

Among the 638 vaccinated, 42.2% (95% CI: 38.3–46.1) expressed an intention to receive the booster dose. The most important reasons given by the participants to receive it included self-protection (51.2%), protecting others (47.5%), and to prevent the next wave (40.1%). In contrast, the most common reasons for not receiving the booster dose included an unwillingness to be forced into taking it (31.2%), a worry that the vaccine would trigger hidden problems in the body (25.7%), and concerns about adverse effects (23.3%) (Supplementary Table 3).

From the LPA, believers (36.8%) predominated followed by fence-sitters (33.5%), apathetics (25.9%), and skeptics (3.8%). Demographic characteristics varied across the profiles (Table 3). Believers were older than fence-sitters and apathetics, and reported better perceived health than apathetics. They were also more likely to have been vaccinated against the seasonal flu than the others. The profiles significantly differed in their mean intention to take the booster dose (*p* < 0.001) with believers having the highest intention (7.64), followed by apathetics (4.98), fence-sitters (4.56), and skeptics (1.58).

**Table 3:** Three-step results for predictors (4 profiles).

	**Vaccination status**															
	**Vaccinated (*****n*** = **638)**								**Unvaccinated** **(n** = **218)**							
**Predictors**	**C1: skeptic** **(*****n*** = **24)**		**C2: believer** **(*****n*** = **235)**		**C3: fence-sitter** **(*****n*** = **214)**		**C4: apathetic** **(*****n*** = **165)**		**C1: skeptic** **(*****n*** = **58)**		**C2: believer** **(*****n*** = **23)**		**C3: Fence-sitter** **(n** = **77)**		**C4: Apathetic** **(n** = **60)**	
	***N***	**%**	***n***	**%**	***n***	**%**	***n***	**%**	***n***	**%**	***N***	**%**	**n**	**%**	**n**	**%**
**Age (years)**		>C3, >C4		< C2		< C2		< C2		>C1, >C3, >C4		< C2		< C2	
18–34	12	50.0%	80	34.0%	99	46.3%	69	41.8%	30	51.7%	7	30.4%	33	42.9%	28	46.7%
35–54	8	33.3%	117	49.8%	93	43.5%	81	49.1%	23	39.7%	8	34.8%	32	41.6%	26	43.3%
55+	4	16.7%	38	16.2%	22	10.3%	15	9.1%	5	8.6%	8	34.8%	12	15.6%	6	10.0%
**Sex**	* < C3		*(< C3)		*>C1, (>C2), >C4		* < C3					
Male	13	54.2%	94	40.0%	64	29.9%	67	40.6%	24	41.4%	7	30.4%	22	28.6%	27	45.0%
Female	11	45.8%	141	60.0%	150	70.1%	98	59.4%	34	58.6%	16	69.6%	55	71.4%	33	55.0%
**Health**			>C4			< C2		>C3			< C1		
Below average	4	16.7%	7	3.0%	3	1.4%	11	6.7%	2	3.4%	1	4.3%	5	6.5%	3	5.0%
Average	4	16.7%	45	19.1%	58	27.1%	47	28.5%	9	15.5%	10	43.5%	29	37.7%	16	26.7%
Above average	16	66.7%	183	77.9%	153	71.5%	107	64.8%	47	81.0%	12	52.2%	43	55.8%	41	68.3%
**Respiratory symptoms in**			>C4				< C2		>C2		< C1, < C3, < C4		>C2		>C2	
**recent 2 weeks**							
No	20	83.3%	208	88.5%	197	92.1%	154	93.3%	52	89.7%	21	91.3%	69	89.6%	54	90.0%
Yes	4	16.7%	27	11.5%	17	7.9%	11	6.7%	6	10.3%	2	8.7%	8	10.4%	6	10.0%
**Smoking status**		>C2		< C1, < C3, < C4		>C2		>C2	
Non-smoker	22	91.7%	220	93.6%	199	93.0%	149	90.3%	54	93.1%	22	95.7%	72	93.5%	55	91.7%
Smoker	2	8.3%	15	6.4%	15	7.0%	16	9.7%	4	6.9%	1	4.3%	5	6.5%	5	8.3%
**Allergies**		(< C2)		(>C1), >C4			< C2	
No	22	91.7%	203	86.4%	180	84.1%	139	84.2%	47	81.0%	17	73.9%	54	70.1%	52	86.7%
Yes	2	8.3%	32	13.6%	34	15.9%	26	15.8%	11	19.0%	6	26.1%	23	29.9%	8	13.3%
**Long-term illness**					
No	13	54.2%	132	56.2%	117	54.7%	101	61.2%	31	53.4%	9	39.1%	37	48.1%	36	60.0%
Yes	11	45.8%	103	43.8%	97	45.3%	64	38.8%	27	46.6%	14	60.9%	40	51.9%	24	40.0%
**Full-time job**		>C2		< C1, < C3, (< C4)		>C2		(>C2)	
No	11	45.8%	102	43.4%	84	39.3%	62	37.6%	21	36.2%	14	60.9%	31	40.3%	27	45.0%
Yes	13	54.2%	133	56.6%	130	60.7%	103	62.4%	37	63.8%	9	39.1%	46	59.7%	33	55.0%
**Adverse effect**					
No	6	25.0%	77	32.8%	63	29.4%	51	30.9%	Not Applicable							
Yes	18	75.0%	158	67.2%	151	70.6%	114	69.1%					
**Flu vaccination last season**	< C2, < C4		>C1, >C3, (>C4)		< C2, < C4		>C1, (< C2), >C3		< C2, < C4		>C1, >C3, >C4		< C2		>C1, < C2	
No	22	91.7%	138	58.7%	169	79.0%	111	67.3%	56	96.6%	14	60.9%	65	84.4%	49	81.7%
Yes	2	8.3%	97	41.3%	45	21.0%	54	32.7%	2	3.4%	9	39.1%	12	15.6%	11	18.3%

Values in the table are estimates from the R3STEP logistic regression analyses using Mplus. We adopted the profile structure of the full sample to the split samples. The resultant profile sizes were slightly different from the full sample. Profile sizes shown in this table are from the full sample.

*The direction indicates the difference in the proportion of women. () p < 0.10.

The profiles significantly differed in their mean intention to take the booster dose (*p* < 0.001) with believers having the highest intention (7.64), followed by apathetics (4.98) and fence-sitters (4.56) while skeptics had the lowest intention (1.58).

From the logistic regression analysis, after adjusting for age, sex, smoking status, previous experience of adverse effects and cohort, three vaccine hesitancy constructs were associated with the intention to receive a booster dose (Table 4). Increased collective responsibility and confidence in the safety and efficacy of vaccines were associated with an increased likelihood to receive a booster dose while increased constraint was associated with a decreased likelihood.

**Table 4:** Factors associated with the intention to receive a third COVID-19 vaccine dose (n = 638).

	**Intention to receive a third COVID-19 dose**		**Adjusted OR** ** (95% CI)**	***p*-value**
**Factor**	**Yes** ** (*n* = 269)**	**No** ** (*n* = 369)**		
**Age group (years)**				
18–34 (reference group)	80 (30.8)	180 (69.2)	
35–44	142 (47.5)	157 (52.5)	1.61 (1.09–2.38)	**0.017**
55+	47 (59.5)	32 (40.5)	1.92 (1.06–3.49)	**0.032**
**Sex**				
Female (reference group)	163 (40.8)	237 (59.2)	
Male	106 (44.5)	132 (55.5)	1.04 (0.71–1.53)	0.822
**Cohort**				
First (R1-R10)	63 (31.7)	136 (68.3)	
Second (R10 “top-up”)	206 (46.9)	233 (53.1)	2.04 (1.36–3.10)	**0.001**
**Smoking status**				
Non-smoker (reference group)	236 (41.0)	340 (59.0)	
Smoker	33 (53.2)	29 (46.8)	2.21 (1.22–4.03)	**0.009**
**Experienced any adverse effects** ^**1**^				
No (reference group)	109 (55.3)	88 (44.7)	
Yes	160 (36.3)	281 (63.7)	0.52 (0.35–0.77)	**0.001**
**Constructs of vaccine hesitancy**^**2**^ **(median, IQR)**				
Confidence	5.3 (4.0–6.0)	4.0 (3.3–5.0)	1.54 (1.30–1.84)	**<0.001**
Complacency	3.3 (2.3–4.0)	4.0 (3.0–4.3)	0.85 (0.71–1.01)	0.070
Constraint	2.7 (2.0–4.0)	3.7 (2.7–4.0)	0.82 (0.69–0.98)	**0.027**
Calculation	5.7 (4.7–6.0)	5.3 (4.3–6.0)	0.94 (0.77–1.14)	0.536
Collective	5.7 (4.7–6.3)	4.7 (4.0–5.7)	1.30 (1.04–1.64)	**0.023**

^1^From the first and/or second COVID-19 vaccine dose.

^2^Average scores of items within each construct were computed, with higher scores indicating higher levels of the constructs. Cronbach's alpha values for the 5 constructs ranged from 0.72 - 0.87.OR,Odds ratio; CI, Confidence interval. Bold figures indicate *p*-values smaller than 0.05.

In the sensitivity analysis, including the six respondents who gave inconsistent responses made little difference to the results.

(iii) Unvaccinated subgroup

As a regression analysis on the intention for the first dose was not statistically powered enough to keep more than two parameters in any model to remain close to the 1 parameter per 8–10 positive guidelines, only LPA was conducted for this subgroup. From the LPA, the majority of the unvaccinated participants were fence-sitters (35.3%), followed by apathetics (27.4%), skeptics (26.6%), and believers (10.6%). The profiles significantly differed in their mean intention for the first vaccine dose (χ^2^ = 64.31, *p* < 0.001) with believers having the highest intention (4.63), followed by fence-sitters (2.88) and apathetics (2.70), while skeptics had the lowest intention (0.89).

## Discussion

### Principal findings

Although almost 75% of the participants had received the COVID-19 vaccine (74.7% received 1 dose, 70.9% received 2 doses), only 42.2% of those who had received or intended to receive two doses had an intention to receive a third dose, which foretells the booster intended uptake lagging initial vaccination. These figures are comparable to those reported by the Hong Kong government in which 76.0% and 70.7% of residents aged 18 years or more had been vaccinated with one or two doses up to 17 January 2022 (6), the cutoff date of our survey.

Our current four profile pattern in the full sample was very similar to the one found in a survey conducted among nurses during the early epidemic phase in March 2020 except that the “contradictor” profile was missing (18). The “middler” and “outsider” profiles defined in our previous work were classified as fence-sitters and apathetics, respectively in this study. The “contradictor” profile was no longer applicable to the general population sample at the time of the survey as the respondents who thought the disease was not as severe as claimed in the earlier wave had a higher risk perception toward Omicron. Believers were shown to have the highest intention to have the booster dose, followed by apathetics, fence-sitters and skeptics.

Factors associated with a person's decision to receive the booster dose included older age, being a smoker, having confidence in vaccines and the authorities who procure and distribute them, and having a sense of collective responsibility. In contrast, having a history of experiencing an adverse effect from previous doses of the vaccine and structural and psychological barriers in accessing vaccination services were associated with a low willingness to receive the booster dose.

### Result implications

Our study has several implications. First, the proportion of participants having an intention to receive the third vaccine dose remained low across vulnerable sub-groups. A recent study revealed that the effectiveness of the vaccine against hospitalization waned among persons aged 65 years or above in a clinically vulnerable group (19) and in persons aged 40 to 64 years with underlying medical conditions than in healthy adults (20). This highlighted the need for a booster dose among these subpopulations. However, the proportion intending to receive a booster dose among individuals aged 45–64, with long-term illnesses, receiving immunosuppressant treatment, and with bad self-perceived health ranged from 44.4 to 53.3%. The latest co-circulation of both Omicron and Delta in Hong Kong will likely result in a substantial hospital burden attributed to a large number of infections from these subgroups if the epidemic is not controlled.

Second, factors associated with COVID-19 vaccination may no longer be associated with the intention to receive the booster dose. Influenza vaccination history was considered an associated factor with increased acceptance of the COVID-19 vaccine. However, it was not associated with COVID-19 revaccination (21). More evidence on the number of booster doses required in the future, rather than their influenza vaccination history, might affect people's current perceptions. The co-administration of both influenza vaccination and a COVID-19 booster dose may not be an option in the short term. Compared to adults aged 18 to 34 years, adults aged 55 years or over were the most willing group followed by individuals aged 35–44 years for receiving a booster dose. This relationship was reversed in a previous study from our group for a different outcome on the intention to receive a COVID-19 vaccine in the same study sample (22). Older people were less likely to receive the COVID-19 vaccine. And when they did, they might be more determined (more likely to be believers) and thus more accepting of the booster dose.

Smoking status was associated with the COVID vaccination intention in this study, unlike in our previous study. Taking off their protective mask for smoking (23) and the increased infection risk attributed to smoking (24) may explain why smokers were more likely to receive a booster dose than non-smokers. To escape from the restrictions imposed by the vaccine bubble policies for unvaccinated individuals, smokers, who are usually lower in health awareness (25), might tend to disregard the risk of potential adverse effects of the booster and could be more motivated to take the booster. The association between smoking and vaccination intention is equivocal in the literature. While the current findings echoed with two other studies conducted in Japan (26) and Egypt (27) in the COVID-19 context, other studies conducted in Western countries (28) and in a non-COVID-19 (29–31) context suggested the opposite. Cultural factors [e.g., power distance (32) and individualism (33)] and contextual factors [e.g., pandemic versus non-pandemic period, trust and psychological reactance in the society (34, 35)] could be potential moderators in the smoking-vaccination linkage. Other psychological factors, including future time perspective (36) and the risk perception of the poorer prognosis (27) brought by smoking, could also be possible candidates to explore. Future endeavors are needed to debunk the equivocality in the association between smoking and vaccination intention. Among the 5Cs, compared with our previous work among local nurses, greater confidence and collective responsibility were similarly independently associated with an increased booster uptake intention. However, unlike in our previous work, complacency was not significant in this study. Instead, having less constraint was a newly found associated antecedent for higher booster intention. Experiencing an adverse effect in previous doses, which was not available in the previous study, was found to impede the intention for the booster. Helping the public to have a greater understanding and management of the potential adverse effects after COVID-19 vaccination may therefore promote a greater acceptance of a booster dose. Delivering free door-to-door vaccination services could be especially beneficial to people with impaired mobility by alleviating the constraints to access the vaccines.

Third, an improvement in vaccination coverage for the first and third doses seems possible in this population. Among vaccinated individuals who had no intention to receive the booster dose, fence-sitters and apathetics accounted for 70% of this subpopulation. Similarly, these two profiles were also the majority (~59%) among unvaccinated without any intention to receive the first dose. Generally, fence-sitters are neutral in their vaccination attitudes while apathetics are not aware or are disinterested. These two profiles with significantly lower intention to be vaccinated than the believers disprove the common misconception that individuals with low interest to be vaccinated are all skeptics or vaccine hesitant (37). Among the unvaccinated, “skeptics” accounted for about 10% of this subpopulation. Although it is challenging to persuade “skeptics”, these individuals did not form a critical mass. To improve the first and third dose vaccination coverage, the government could adopt strategies to help sway fence-sitters (38, 39) and persuade apathetic individuals by rebuilding their trust in the health authorities (40, 41) and providing additional information to drive personal benefits and costs (42). The government's plan to expand the implementation of a vaccine pass, which requires teachers and school staff to provide proof of vaccination to access schools and other groups to access catering and leisure facilities, would likely be a strong incentive. More work should be done to have an optimal plan for vaccine supply by examining the increased demand for heterologous boosting among vaccinated (5.2% switched from Sinovac to BioNTech as their intended or actual vaccine type, Supplementary Table 4) and vaccination due to the implementation of a COVID-19 certification (43).

Finally, discussing both a person-centered and variable-centered approach together to dissect ways to enhance vaccination together may provide a more thorough understanding of each (44). To our best knowledge, although many studies around the world (45, 46) have examined factors related to vaccine hesitancy (or intention), this is the first study to identify associated factors and profiles of COVID-19 revaccination to respond to the interim statement on booster doses for COVID-19 vaccination.

### Limitations

Our study has some limitations. First, the major outcome was the revaccination intention, not the actual decision, although several studies have suggested a positive correlation between intention and behavior (47). Given the abundant supply of COVID-19 vaccines and easy access to vaccination services among the general public, discrepancies between intention and behavior may be minimal (48). Future follow-up studies estimating the proportion of individuals who acted accordingly could address these variations. Second, our study population was limited to adults living in Hong Kong. Third, our online survey could be more difficult to access for older people or those who have low proficiency in the use of mobile devices and internet technology, thus the cohort could be potentially less representative of the older population. Consequently, we are not able to provide suggestions to refine existing strategies to improve the current vaccination coverage among individuals aged between 5 and 17 years. Fourth, causal relationships cannot be established from a cross-sectional analysis.

### Conclusions

Given the fourth COVID-19 vaccine dose may offer to all adults, this study helps to refine existing and future strategies in vaccination campaigns. Strategies for improving boosting uptake include targeting the younger adults, providing additional information to alleviate concerns about the vaccine's adverse effects, improving confidence in vaccines by the general public, rebuilding the trust of fence-sitters in the health authorities (40, 41) and providing apathetics additional information to drive personal benefits and costs.

## Data availability statement

The raw data supporting the conclusions of this article will be made available by the authors, without undue reservation.

## Ethics statement

The studies involving human participants were reviewed and approved by Survey Behavioral Research Ethics Committee of the Chinese University of Hong Kong (reference number: SBRE-20-037). The patients/participants provided their written informed consent to participate in this study.

## Author contributions

Conceptualization: KK, KL, and SW. Formal analysis: KK, KL, CL, and EM. Methodology: KK, KL, CL, EM, AT, EC, and WW. Writing-original draft: KK. Data collection: WW and MT. Writing-review and editing: KL, CL, AT, EC, MT, and EM. Supervision: SW. All authors critically assessed the final version of the submitted manuscript. All authors contributed to the article and approved the submitted version.

## Funding

The Group Research Scheme of the Chinese University of Hong Kong provided support on the incentives to the participants of the online survey.

## Conflict of interest

The authors declare that the research was conducted in the absence of any commercial or financial relationships that could be construed as a potential conflict of interest.

## Publisher's note

All claims expressed in this article are solely those of the authors and do not necessarily represent those of their affiliated organizations, or those of the publisher, the editors and the reviewers. Any product that may be evaluated in this article, or claim that may be made by its manufacturer, is not guaranteed or endorsed by the publisher.
