# Supplementary material for: The way forward to achieve high COVID-19 vaccination and revaccination coverage in a city amid a period of tranquility
Source: Front Public Health. 2022 Sep 14;10:935243. doi: 10.3389/fpubh.2022.935243 (PMC9515959; doi:10.3389/fpubh.2022.935243)
Supplement: Supplementary file 1 [file Data_Sheet_1.docx]

**Appendix**

*Descriptive results*

Subjects were compensated with shopping/gift vouchers valued at HKD$60. The total number of people approached was 1,895 of which 890 completed the online survey (completion rate = 47.0%). Of these, 28 were excluded for the following reasons: 17 were aged less than 18 years; five were non-residents; six gave invalid responses (no variation in the 5C vaccine hesitancy items with conflicting responses). There were 862 eligible subjects in this study. A flow diagram outlining the sequence of questions on vaccination behavior and intention is shown in Figure S1.

**i) Full sample**

Among 856 responses from individuals who did not receive the vaccine (n=218) and those receiving or intending to receive two doses (n=638), there were 318 males and 538 females and the majority (62.0%) were aged between 25 and 44 years. Most (58.9%) had full-time jobs and perceived their health as being “good” (55.8%) or “very good” (14.5%). Long-term illnesses were reported by 380 (43.1%) participants, the most common ones being ear/nose/throat conditions (n=124), high blood pressure (n=69) and kidney disease (n=63). Food or drug allergies were reported by 142 (5.6%) participants and 17 had ever been treated with immunosuppressants. Six participants had a history of organ transplant.

**ii) Vaccinated subgroup**

Among the 638 vaccinated, 269 (42.2%, 95% CI: 36.3 - 48.1) had received (n=27) or intended to receive (n=242) the booster dose. The majority were females (n=400, 62.7%) and were aged between 25 and 44 years (n= 393, 61.6%). About 62.7% of this sub-population had full-time jobs and 71.9% rated their health as “good” or “very good”. Long-term illnesses were reported by 275 subjects (43.1%). The most common ones being ear/nose/throat conditions (n=89), hypertension (n=50) and kidney disease (n=47). Food or drug allergies were reported by 94 (14.7%) and only 10 (1.6%) had ever been treated with immunosuppressants. Three participants had a history of organ transplant. Self-protection (51.2%) and protecting others (47.5%) were the most common reasons given while an unwillingness to be forced into taking it (31.2%) and a worry that the vaccine would trigger hidden problems in the body (25.7%) were common reasons for not receiving the vaccine (Appendix Table S3).

. .

**iii) Unvaccinated subgroup**

Among the 218 unvaccinated, 19 (8.7%, 95% CI: 5.3 - 13.3) expressed an intention to take the first dose. In this subpopulation, there were 80 males and 138 females and most (63.3%) were aged between 25 and 44 years. Most (57.3%) had full-time jobs and reported having a “good” (50.9%) or “very good” (14.7%) level of health. Long-term illnesses were reported by 105 (48.2%), the most common ones being ear/nose/throat conditions (n=35), hypertension (n=19) and skin conditions (n=17). Food or drug allergies were reported by 48 (22.0%) and only 7 had ever been treated with immunosuppressants. Three had a history of organ transplant.

The majority of the unvaccinated participants were fence-sitters (35.3%), followed by apathetics (27.4%), skeptics (26.6%), and believers (10.6%). Believers were older than skeptics, fence-sitters, and apathetics, were less likely to report respiratory symptoms in the past two weeks and be smokers than skeptics, fence-sitters, and apathetics, and were more likely to report having allergies than apathetics. They were more likely to have been vaccinated against the seasonal flu than the other three profiles. Fence-sitters were more likely to have worse perceived health than skeptics while apathetics were more likely to have been vaccinated against the seasonal flu compared to skeptics

*Comparison of participants recruited in the existing cohort and top-up cohort.*

We conducted a separate analysis to investigate the potential influence of the participants in the top-up cohort in the data analysis. Those recruited in the top-up cohort were similar in terms of distribution of gender, smoking status, and self-reported health. However, this cohort was significantly older, had a higher proportion of full-time employees, a higher proportion who received the influenza vaccination, and a lower proportion who experienced adverse effects from the COVID-19 vaccine.

*Model selection in the Latent Profile Analysis*

Constructs of the 5C scale are conceptually different in their associations with the vaccination intention (confidence and collective responsibility being positive; complacency, constraint, and calculation being negative,[[1]](https://paperpile.com/c/WXB5QR/W4J8f)). It is also suggested that response set bias could distort the profile structure in the latent profile analysis (e.g.,[[2]](https://paperpile.com/c/WXB5QR/Ip3MV)).

Fitted statistics of latent profile analysis such as LL, AIC, BIC and sBIC decreased as the profile number increased with significant BLRT until the profile number reached 18 (Table S3). The selection of the profile pattern was informed by the fit statistics and prior findings. We picked the 4-profile solution as it outperformed the *k-1* solution (i.e., the 3-profile solution) with the significant BLRT (but not LMR) and provided a more insightful profile pattern than the 3-profile solution (with a qualitatively different profile, the “apathetic”, introduced on top of the three quantitatively different profiles, namely, the “skeptic”, the “believer”, and the “fence-sitter”. The 4-profile solution maintained the smallest profile size at more than 5% of the full sample (with the smallest being the “skeptic”, at the size of 9.58% of the total sample), a threshold deemed favorable to avoid spurious fragmented profiles[[3,4]](https://paperpile.com/c/WXB5QR/xI4vl+FTtcN). Solutions with more than 4-profile were, therefore, not further examined, as there were fragmented profiles with the size less than 5% of the total sample and less than 30 members. The 4-profile solution was also substantially consistent with the profiles of vaccine hesitancy found in a nurse sample[[5]](https://paperpile.com/c/WXB5QR/Y7TTI).

We used the R3STEP and DCON commands of Mplus to model the predictors and vaccination intentions, respectively, for two subgroups - one for those who were unvaccinated and another for those who were vaccinated (received 2 doses or received 1 dose but intended to received 2 doses).

Reference

[1] [Betsch C, Böhm R, Chapman GB. Using Behavioral Insights to Increase Vaccination Policy Effectiveness. Policy Insights from the Behavioral and Brain Sciences 2015;2:61–73.](http://paperpile.com/b/WXB5QR/W4J8f)

[2] [Geiser C, Koch T, Eid M. Data-generating mechanisms versus constructively-defined latent variables in multitrait-multimethod analysis: A comment on Castro-Schilo, Widaman, and Grimm (2013). Struct Equ Modeling 2014;21:509–23.](http://paperpile.com/b/WXB5QR/Ip3MV)

[3] [Marsh HW, Lüdtke O, Trautwein U, Morin AJS. Classical Latent Profile Analysis of Academic Self-Concept Dimensions: Synergy of Person- and Variable-Centered Approaches to Theoretical Models of Self-Concept. Struct Equ Modeling 2009;16:191–225.](http://paperpile.com/b/WXB5QR/xI4vl)

[4] [Ferguson SL, G. Moore EW, Hull DM. Finding latent groups in observed data: A primer on latent profile analysis in Mplus for applied researchers. Int J Behav Dev 2020;44:458–68.](http://paperpile.com/b/WXB5QR/FTtcN)

[5] [Leung CLK, Li K-K, Wei VWI, Tang A, Wong SYS, Lee SS, et al. Profiling vaccine believers and skeptics in nurses: A latent profile analysis. Int J Nurs Stud 2022;126:104142.](http://paperpile.com/b/WXB5QR/Y7TTI)

**Appendix tables**

| Table S1. Fitted statistics for profile structures (N =856) | | | | | | | | | |
| --- | --- | --- | --- | --- | --- | --- | --- | --- | --- |
| Number of profiles | LL | FP | AIC | BIC | sBIC | LMR (*p*) | BLRT (*p*) | Entropy | Smallest class % |
| 1 | -6964.987 | 10 | 13949.973 | 13997.496 | 13965.739 | NA | NA | NA | NA |
| 2 | -6653.483 | 16 | 13338.966 | 13415.001 | 13364.191 | <.0000 | <.0000 | .755 | 42.17% |
| 3 | -6526.796 | 22 | 13097.593 | 13202.143 | 13132.277 | .0004 | <.0000 | .827 | 7.83% |
| 4 | -6478.054 | 28 | 13012.108 | 13145.171 | 13056.251 | .1352 | <.0000 | .742 | 9.58% |
| 5 | -6434.837 | 34 | 12937.675 | 13099.252 | 12991.278 | .2986 | <.0000 | .795 | 1.75% |
| 6 | -6390.657 | 40 | 12861.314 | 13051.405 | 12924.376 | .2039 | <.0000 | .823 | 0.94% |
| 7 | -6350.809 | 46 | 12793.618 | 13012.222 | 12866.139 | .5306 | <.0000 | .814 | 0.94% |
| 8 | -6318.010 | 52 | 12740.021 | 12987.139 | 12822.001 | .1556 | <.0000 | .821 | 0.94% |
| 9 | -6286.912 | 58 | 12689.825 | 12965.456 | 12781.264 | .2478 | <.0000 | .821 | 0.94% |
| 10 | -6260.803 | 64 | 12649.606 | 12953.751 | 12750.505 | .6161 | <.0000 | .822 | 0.94% |
| 11 | -6234.808 | 70 | 12609.615 | 12942.274 | 12719.974 | .1363 | <.0000 | .834 | 0.94% |
| 12 | -6211.457 | 76 | 12574.914 | 12936.087 | 12694.732 | .1358 | <.0000 | .839 | 0.47% |
| 13 | -6193.297 | 82 | 12550.594 | 12940.280 | 12679.871 | .3587 | <.0000 | .838 | 0.47% |
| 14 | -6180.749 | 88 | 12537.498 | 12955.698 | 12676.235 | .5134 | <.0000 | .839 | 0.12% |
| 15 | -6158.320 | 94 | 12504.640 | 12951.353 | 12652.835 | .5003 | <.0000 | .839 | 0.47% |
| 16 | -6138.828 | 100 | 12477.856 | 12952.883 | 12635.111 | .4685 | <.0000 | .848 | 0.12% |
| 17 | -6122.003 | 106 | 12456.006 | 12959.746 | 12623.120 | .3920 | <.0000 | .856 | 0.12% |
| 18 | -6109.235 | 112 | 12442.470 | 12974.724 | 12619.044 | .7282 | .1429 | .844 | 0.23% |
| *Notes.* LL = log-likelihood; FP = free parameters; AIC = Akaike information criteria; BIC = Bayesian information criteria; sBIC = sample-size- adjusted BIC; LMR = Vuong-Lo-Mendell-Rubin likelihood ratio test; BLRT = bootstrapped likelihood ratio test. | | | | | | | | |  |

| Table S2. Modelling predictors using logistic regression (N = 856) | | | | | | | | |
| --- | --- | --- | --- | --- | --- | --- | --- | --- |
| **Predictor** | **Skeptic (C1)**  **(n = 82)** | | **Believer (C2)**  **(n = 258)** | | **Fence-sitter (C3)**  **(n = 291)** | | **Apathetic (C4)**  **(n = 225)** | |
|  | **n** | **%** | **n** | **%** | **n** | **%** | **n** | **%** |
| **Age** | <C2 | | >C1, >C3, >C4 | | <C2 | | <C2 | |
| 18-34 | 42 | 51.2% | 87 | 33.7% | 132 | 45.4% | 97 | 43.1% |
| 35-54 | 31 | 37.8% | 125 | 48.4% | 125 | 43.0% | 107 | 47.6% |
| 55+ | 9 | 11.0% | 46 | 17.8% | 34 | 11.7% | 21 | 9.3% |
| **Sex** | *<C3 | | *<C3 | | *>C1, >C2, >C4 | | *<C3 | |
| Male | 37 | 45.1% | 101 | 39.1% | 86 | 30.6% | 94 | 41.8% |
| Female | 45 | 54.9% | 157 | 60.9% | 205 | 70.4% | 131 | 58.2% |
| **Perceived health** |  |  | >C3, >C4 | | <C2 | | <C2 | |
| Below average | 6 | 7.3% | 8 | 3.10% | 8 | 2.7% | 14 | 6.2% |
| Average | 13 | 15.9% | 55 | 21.3% | 87 | 29.9% | 63 | 28.0% |
| Above average | 63 | 76.8% | 195 | 75.6% | 196 | 67.4% | 148 | 65.8% |
| **Flu vaccination last season** | <C2, (<C3), <C4 | | >C1, >C3, >C4 | | (>C1), <C2, <C4 | | >C1, <C2, >C3 | |
| Yes | 4 | 4.9% | 106 | 41.1% | 57 | 19.6% | 65 | 28.9% |
| No | 78 | 95.1% | 152 | 58.9% | 234 | 80.4% | 160 | 71.1% |
| **Taken two doses of COVID-19 vaccines** | <C2, <C3, <C4 | | >C1, >C3, >C4 | | >C1, <C2 | | >C1, <C2 | |
| Yes | 24 | 29.3% | 235 | 91.1% | 214 | 73.5% | 165 | 73.3% |
| No | 58 | 70.7% | 23 | 8.9% | 77 | 26.5% | 60 | 26.7% |
| Values in the table are estimates from the R3STEP function of Mplus. We have also examined having respiratory symptoms in the recent two weeks, reported having long-term illnesses, with full-time job, and experienced adverse effects in their first and/or second dose in the R3STEP function, none of them yielded significant difference across profiles.  *The direction indicates the difference in the proportion of women.  ( ) *p* < .10. | | | | | | | | |

### Table S3. Importance^1^ of various reasons for receiving or not receiving the COVID-19 vaccine

|  | **Mean**  **(SD)** | **Most**  **n (%)** |
| --- | --- | --- |
| **A. Reasons for deciding to receive the COVID-19 vaccine (N=638)** |  |  |
| To protect myself from getting infected | 3.88 (0.98) | 176 (27.6) |
| To protect others from getting infected | 3.86 (1.02) | 181 (28.4) |
| Job requirements | 3.79 (1.08) | 187 (29.3) |
| To prevent the next wave of the outbreak | 3.57 (1.21) | 147 (23.0) |
| For outbound trip | 3.55 (1.21) | 152 (23.8) |
| To resume the normality | 3.51 (1.29) | 167 (26.2) |
| For entering restaurants and other entertainment venues | 3.32 (1.17) | 92 (14.4) |
| To accelerate economic recovery | 3.22 (1.30) | 119 (18.7) |
| Social responsibility | 3.13 (1.17) | 66 (10.3) |
| Pressure from society | 2.53 (1.16) | 35 (5.5) |
| Pressure from family | 2.42 (1.11) | 26 (4.1) |
| Encouragement from the government | 2.37 (1.29) | 48 (7.5) |
| Incentives given by corporations | 2.36 (1.23) | 40 (6.3) |
| Incentives given by the government | 2.34 (1.24) | 42 (6.6) |
| Pressure from friends | 2.34 (1.09) | 23 (3.6) |
| **B. Reasons for deciding not to receive the COVID-19 vaccine (n=218)** |  |  |
| I worry about the adverse effects of the vaccine | 4.40 (0.84) | 112 (51.4) |
| I worry that the vaccine would trigger hidden problems in my body | 4.35 (0.90) | 110 (50.5) |
| I don't want to be forced into taking the vaccine | 4.14 (1.04) | 96 (44.0) |
| I have doubts about the safety and efficacy statistics given by vaccine companies | 4.09 (0.89) | 77 (35.3) |
| I have doubts about the safety of the vaccine produced using new technologies | 4.06 (0.96) | 78 (35.8) |
| The risk of adverse effects from taking the vaccine is larger than the risk of getting infected | 3.98 (0.99) | 72 (33.0) |
| I don't think the protective effect of the vaccine can last long | 3.92 (0.93) | 56 (25.7) |
| I don't think my bodily situation is suitable for receiving the vaccine | 3.90 (1.01) | 69 (31.7) |
| I don't think the vaccines are effective | 3.82 (0.93) | 54 (24.8) |
| My doctor did not recommend me to take it | 3.68 (1.17) | 54 (24.8) |
| The local outbreak is not severe | 3.53 (1.01) | 42 (19.3) |
| The risk of infection by COVID-19 is low even if I don't get vaccinated | 3.34 (1.04) | 27 (12.4) |
| I'm against vaccination in general | 3.24 (1.04) | 32 (14.7) |
| I prefer natural immunity over vaccine immunity | 3.23 (1.13) | 36 (16.5) |
| The global outbreak is not severe | 3.01 (0.95) | 17 (7.8) |
| Pressure from friends, family and society | 2.66 (1.04) | 5 (2.3) |
| **C. Reasons for deciding to receive the third dose (n=269)** |  |  |
| To protect myself from getting infected | 4.34 (0.82) | 136 (50.6) |
| To protect others from getting infected | 4.28 (0.87) | 130 (48.3) |
| To prevent the next wave of outbreak | 4.13 (0.98) | 111 (41.3) |
| To resume the normality | 4.00 (1.08) | 106 (39.4) |
| To accelerate economic recovery | 3.80 (1.18) | 88 (32.7) |
| For outbound trip | 3.70 (1.17) | 79 (29.4) |
| Job requirement | 3.65 (1.19) | 76 (28.3) |
| For entering restaurants and other entertainment venues | 3.64 (1.17) | 69 (25.7) |
| Social responsibility | 3.59 (1.13) | 57 (21.2) |
| Encouragement from the government | 2.82 (1.39) | 42 (15.6) |
| Pressure from society | 2.66 (1.22) | 25 (9.3) |
| Pressure from the family | 2.64 (1.19) | 22 (8.2) |
| Incentives given by the government | 2.61 (1.29) | 29 (10.8) |
| Incentives given by other corporations | 2.61 (1.30) | 29 (10.8) |
| Pressure from friends | 2.53 (1.17) | 18 (6.7) |
| **D. Reasons for deciding not to receive the third dose (n=369)** |  |  |
| I don't want to be forced into taking the vaccine | 3.82 (1.08) | 115 (18.8) |
| I worry that the vaccine would trigger hidden problems in my body | 3.78 (1.06) | 95 (15.5) |
| I worry about the adverse effects of the vaccine | 3.75 (1.05) | 86 (14.1) |
| I don't think the protective effect of the vaccine can last long | 3.68 (1.03) | 72 (11.8) |
| The risk of adverse effects from taking the vaccine is larger than the risk of getting infected by COVID-19 | 3.56 (1.07) | 68 (11.1) |
| I have encountered adverse effects when taking the first two doses | 3.47 (1.06) | 51 (8.3) |
| I don't think vaccines are effective | 3.47 (1.09) | 62 (10.1) |
| The local outbreak is not severe | 3.41 (1.12) | 60 (9.8) |
| I don't think my bodily situation is suitable for receiving the vaccine | 3.38 (1.11) | 55 (9.0) |
| My doctor advised me against taking it | 3.34 (1.21) | 58 (9.5) |
| Taking two doses of the COVID-19 vaccine would be enough to protect myself | 3.26 (1.01) | 28 (4.6) |
| The global outbreak is not severe | 2.94 (1.00) | 22 (3.6) |
| I'm not in a priority group | 2.83 (1.13) | 21 (3.4) |
| I have been infected by COVID-19 | 2.37 (1.06) | 6 (1.0) |
| Pressure from friends, family and society | 2.37 (1.03) | 6 (1.0) |

**^1^** Participants were asked to rate the importance of each reason on a scale of 1 to 5 where 1=least important and 5=most important. SD: standard deviation.

### Table S4. Choice of vaccines for first and third doses

**A. First dose choice among those who received one and two doses of the vaccine (n=638)**

| **Vaccine type** | **One dose (n=644)** | |  | **Two doses (n=638)** | |
| --- | --- | --- | --- | --- | --- |
|  | **n** | **%** |  | **n** | **%** |
| BioNTech | 549 | 85.2 |  | 544 | 85.3 |
| SinoVac | 95 | 14.8 |  | 94 | 14.7 |

**B. Third dose choice among those who received or intended to receive two doses (n=269)**

|  | **Received (n=27)** | | **Intended (n=242)** | | **Total (n=269)** | |
| --- | --- | --- | --- | --- | --- | --- |
| **Vaccine type** | **n** | **%** | **n** | **%** | **n** | **%** |
| BioNTech | 18 | 66.7 | 201 | 83.1 | 219 | 81.4 |
| SinoVac | 9 | 33.3 | 39 | 16.1 | 48 | 17.8 |
| Moderna | 0 | 0 | 2 | 0.8 | 2 | 0.7 |

**C. Distribution of vaccine choice for first and third dose (n=269)**

| **First dose**  **(received)** | **Third dose**  **(received + intended)** | **n** | **%** |
| --- | --- | --- | --- |
| BioNTech | BioNTech | 205 | 76.2 |
| SinoVac | SinoVac | 47 | 17.5 |
| SinoVac | BioNTech | 14 | 5.2 |
| BioNTech | Moderna | 2 | 0.7 |
| BioNTech | SinoVac | 1 | 0.4 |

**Figures**

**
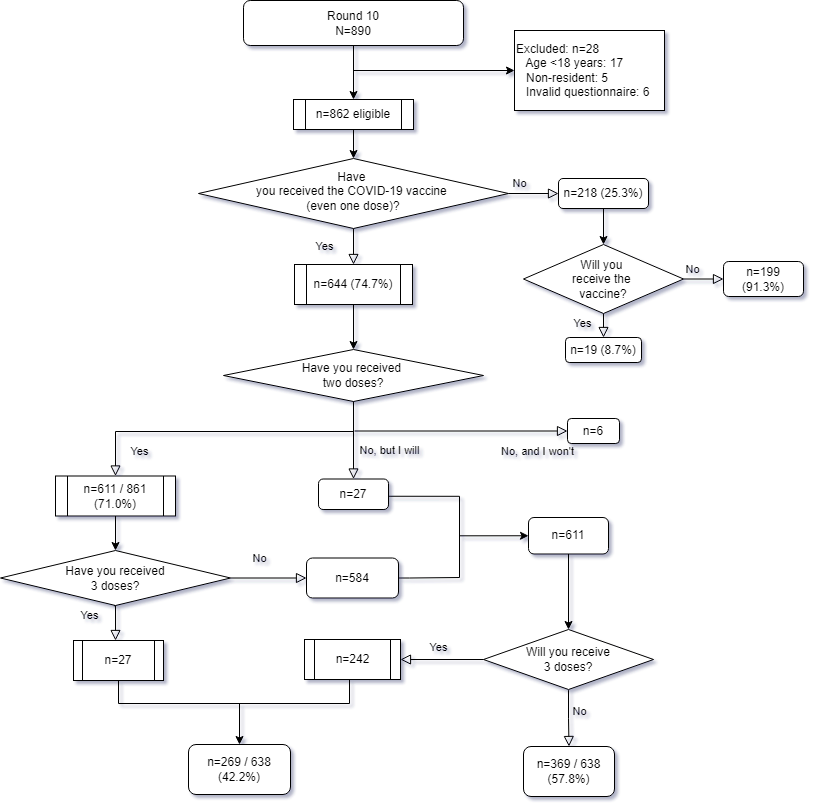
**

### Figure S1. Flow diagram outlining the sequence of questions on vaccination intention and behavior asked to all recruited participants.

### 
